# Intention to leave work among nursing professionals at three university hospitals in Brazil

**DOI:** 10.1590/0034-7167-2025-0192

**Published:** 2026-07-06

**Authors:** Herica Silva Dutra, Diego Marques de Oliveira, Adrize Rutz Porto, Maria Cristina Soares Rodrigues, Diana Albuquerque Alvim de Paula, Paula Roberta Silva Araújo Machado, Lucas da Silva Dellalibera, Nádia Fontoura Sanhudo

**Affiliations:** I Universidade Federal de Juiz de Fora. Juiz de Fora, Minas Gerais, Brazil; II Universidade Federal de Pelotas. Pelotas, Rio Grande do Sul, Brazil; III Universidade de Brasília. Brasília, Distrito Federal, Brazil

**Keywords:** Nursing, Intention, Employment, Personnel Turnover, Job Satisfaction., Enfermería, Intención, Empleo, Reorganización del Personal, Satisfacción en el Trabajo.

## Abstract

**Objectives:**

to analyze factors associated with intention to leave work among nursing professionals at three federal university hospitals in Brazil.

**Methods:**

this was a cross-sectional multicenter study. Data were collected between October 2018 and September 2022, including personal and work-related data, job satisfaction, and intention to leave work. Chi-square, Mann-Whitney, and logistic regression tests were used.

**Results:**

the study included 429 nursing professionals with a mean age of 38.9 years, predominantly female (80%), and nursing technicians (66.2%). Those with higher education or graduate education showed a greater intention to leave their jobs (p=0.029), dissatisfied with their job (p<0.001), who considered staff size inadequate (p=0.026), younger (p=0.014) and with less experience (p=0.046). Low job satisfaction was associated with a three times greater likelihood of leaving the job (OR=3.519; CI=1.594-7.766; p=0.002).

**Conclusions:**

dissatisfied nursing professionals are more likely to intend to leave their jobs.

## RESUMEN

**Objetivos:**

analizar los factores asociados a la intención de abandonar el empleo entre profesionales de enfermería de tres hospitales universitarios federales de Brasil.

**Métodos:**

estudio transversal multicéntrico. Se recopilaron datos entre octubre de 2018 y septiembre de 2022, incluyendo datos personales y laborales, satisfacción laboral e intención de abandonar el empleo. Se utilizaron las pruebas de chi-cuadrado, Mann-Whitney y regresión logística.

**Resultados:**

participaron 429 profesionales de enfermería con una edad media de 38,9 años, predominantemente mujeres (80%) y técnicos de enfermería (66,2%). Aquellos con estudios superiores o títulos de posgrado (p=0,029), insatisfacción laboral (p<0,001), quienes consideraban inadecuado el tamaño del equipo (p=0,026), los más jóvenes (p=0,014) y quienes tenían menos experiencia (p=0,046) mostraron una mayor intención de abandonar sus puestos de trabajo. La baja satisfacción laboral se asoció con una probabilidad tres veces mayor de abandonar el trabajo (OR=3,519; IC=1,594-7,766; p=0,002).

**Conclusiones:**

los profesionales de enfermería insatisfechos tienen mayor probabilidad de querer abandonar sus puestos de trabajo.

## INTRODUCTION

Patient care quality can be directly reflected in healthcare institutions’ ability to retain its employees, especially nursing professionals, who spend the most time in contact with patients^(1)^. Globally, the shortage of nursing professionals was estimated at 6 million in the pre-pandemic period, with projections reaching 13 million in the coming years as a consequence of an increased demand for care and the lack of policies to retain these professionals^(2)^. However, the International Council of Nurses highlights even more alarming figures, estimating a global shortage of up to 30.6 million nursing professionals^(3)^.

Considering this scenario and these trends, the United Nations has outlined the Sustainable Development Goals (SDGs), the third of which aims to “ensure healthy lives and promote well-being for all at all ages”^(4)^. For this to happen, it will be necessary to “substantially increase health financing and the recruitment, development, training and retention of the health workforce in developing countries”^(5)^. It is noteworthy that nursing professionals represent the largest workforce in healthcare. Therefore, achieving health for all is directly related to nursing’s ability to reach its full potential and contribute effectively to global health. This can be done through the application of their knowledge, values, and practical skills, enabling them to respond to current and future healthcare demands^(3)^.

In the Americas, due to existing inequalities among countries, several factors have contributed to the shortage of nurses and nursing technicians, such as migration and labor mobility, poor distribution of healthcare professionals, low professional valuation and recognition, deficiencies in professional training^(6)^, among others. Going against the global trend, Brazil has 70% of its health workforce represented by nurses, with a projected increase to 51% by 2030. However, the unequal professional distribution in different locations, coupled with a lack of worker retention policies, has contributed to increased dissatisfaction with the profession and intention to leave it^(7)^.

Intention to leave work can be understood as the result of a long process of recurring exposure to stressful factors in work environments, increasing job dissatisfaction, emotional detachment from the role, and the feeling of wanting to leave the job as a way of coping with the stressor^(8-10)^. Among the factors influencing intention to leave work in hospital settings are excessive workload, quantitative and qualitative nursing staff inadequacy, emotional exhaustion, interprofessional conflict, demands for physically demanding activities, and workplace violence. The nursing practice environment can also be unfavorable, considering nurse management and leadership styles, lack of empowerment of nursing professionals, lack of support, poor organizational climate, absence of career plans, job dissatisfaction, and little social and professional recognition^(8,9)^.

Although intention to leave work is a well-documented and worrying phenomenon in developed countries^(1)^, Brazilian studies’ contribution on this topic is small when compared to other countries, as identified in an extensive systematic review that assessed 345 studies on the prevalence and determinants of retention of nursing professionals^(1)^. Furthermore, no previous Brazilian multicenter studies discussing intention to leave work and profession were identified. It is also noteworthy that, in Brazil, the loss of qualified professionals to the international market, coupled with dissatisfaction with the profession, may contribute to increased vulnerability of the Brazilian healthcare system^(6)^. These factors, coupled with projections of a global shortage of nursing professionals in the coming years, justify the relevance of this investigation.

## OBJECTIVES

To analyze factors associated with intention to leave work among nursing professionals at three federal university hospitals in Brazil.

## METHODS

### Ethical aspects

This research was submitted for review by the Research Ethics Committees of the three participating institutions. The precepts of Resolution 466/12 of the Brazilian National Ethics Council were respected. The data were obtained after obtaining participant consent, through express agreement in the Informed Consent Form (ICF).

### Study design, period, and location

This is a multicenter, cross-sectional study with a quantitative approach, described in this article in accordance with STrengthening the Reporting of OBservational studies in Epidemiology (STROBE). The study involved three federal university hospitals characterized as medium-sized general hospitals, managed by the *Empresa Brasileira de Serviços Hospitalares*. The participating hospitals are located in three Brazilian regions: Midwest, Southeast, and South. In order to preserve setting anonymity, the institutions will be referred to as hospitals A, B, and C.

Data collection occurred at different times and in different formats across the three settings due to restrictions imposed by the COVID-19 pandemic. Furthermore, ethical review in the settings involved took place at different times due to variations in documentary and bureaucratic procedures, and the prioritization of COVID-19-related studies during the pandemic.

At hospital A, data was collected between October 2018 and April 2019. The invitation was made in person by the researchers, as was the delivery and signing of the printed ICF. The research questionnaire was completed on an Android electronic device provided by the researchers.

At hospital B, data collection took place between November 2020 and June 2021. The invitation was made in person, and the printed ICF was given to participants. The survey form was made available via an electronic link through a messaging application (WhatsApp^®^), and the responses were completed on participants’ own devices.

At hospital C, data was collected between July 2021 and September 2022. The ICF and the link to the survey form were sent to participants via institutional email. Due to low participation in this method, after a change in the epidemiological setting, in-person contact with participants was authorized for data collection. Thus, the ICF and the link to the form were made available directly to participants via messaging application (WhatsApp^®^), which facilitated obtaining responses.

The electronic survey form was made available through the KoBoToolbox software^(11)^. This is a free data collection tool that can be used remotely or in person, with or without internet access.

It is worth noting that, despite the distinct approach to invitation and access to the electronic form with the data collection instrument, all participants responded to the same standardized form made available through the KoBoToolbox software. All participants had the opportunity to clarify their doubts regarding the form completion, and there was no time limit to complete the answers.

### Population or sample; inclusion and exclusion criteria

The total number of 429 participants was calculated considering the power of the statistical test^(12)^. Using the G*Power 3.1.9.3 software, the sample size was determined using the mean effect size and the possibility of type I errors. (α = 0.05) and II (β = 0.80). Thus, a sample of 143 participants was obtained in each setting considering Pearson’s chi-square test.

Participants met the following inclusion criteria for the study: a) being a nurse or nursing technician; b) having been employed for at least three months at the institution targeted by the research (due to the three-month probationary period); c) not being on vacation or leave at the time of data collection. The exclusion criterion was: a) performing exclusively administrative or managerial activities (due to the interest in the care staff’s perspective). At hospitals A and C, a stratified random sample with replacement was adopted for nurses and nursing technicians. Selection was made from a list provided by nursing management. At hospital B, a convenience sample was adopted.

### Study protocol

For this study, the variables listed in the “general information” section of MISSCARE-*BRASIL* were adapted. MISSCARE was developed in English^(13)^ and subsequently validated in Brazil, with the inclusion of items that better reflected the Brazilian reality^(14)^. In this study, only variables related to personal and work characteristics were considered, in addition to job satisfaction and intention to leave nursing.

Characterization variables included sex (male/female), age (in years), position (nurse/nursing technician), highest educational level (high school/higher education/graduate education), years of experience in the position, years of experience in the unit (in years), work period (day/night/rotating shifts), number of patients seen on the current shift, and frequency with which they feel the number of staff in their unit/sector is inadequate (0%, 25%, 50%, 75%, and 100% of the time).

Intention to leave nursing was assessed using the question “Do you plan to leave your current position or role?”, with the answer options “in the next six months”, “in the next year” and “I do not plan to leave my current position/role”. Subsequently, job satisfaction was quantified using the question “How satisfied are you in your current position/role?”, with the answer options “very satisfied”, “satisfied”, “neither satisfied nor dissatisfied”, “dissatisfied” or “very dissatisfied”.

### Analysis of results and statistics

The data were tabulated using Microsoft Excel for Windows^®^. Frequency tables (absolute and percentage) were created to describe the sample characteristics. Measures of position of continuous variables were obtained (mean, median, standard deviation, quartiles, maximum value, and minimum value).

For data analysis purposes, responses regarding job satisfaction were grouped as follows: responses such as “very satisfied” and “satisfied” were categorized as “satisfied”; and responses such as “neither satisfied nor dissatisfied”, “very dissatisfied”, and “dissatisfied” were categorized as “dissatisfied”. Similarly, for the “intention to leave nursing” variable, responses such as “in the next six months” and “in the next year” were grouped as “yes”. The “frequency with which you feel the number of employees in your unit/sector is inadequate” variable was grouped as “greater than or equal to 50% of the time” and “less than 50% of the time”.

Intention to leave nursing was a dependent variable, and the others were independent variables. In order to minimize possible biases, the following procedures were adopted: availability to clarify doubts for participants throughout the development of this study; guarantee of anonymity in dissemination of results; no time limit for completing the data collection form; and careful and impartial analysis of the data.

The association among categorical variables was verified using Pearson’s chi-square test. The relationship between continuous variables and intention to leave work was verified using the Mann-Whitney test, considering the result of the normality test of quantitative variables. To assess the association of independent variables with the “intention to leave work” dichotomized dependent variable (yes/no), logistic regression was performed. The Enter method was used, and the Hosmer & Lemeshow test was considered to verify the fit of the final model (*X* ^2^=9.562; p=0.297). Statistical analyses were performed using the Statistical Package for the Social Sciences version 30. A p-value <0.05 was considered for the statistical tests employed.

## RESULTS

A total of 429 nursing professionals participated, 143 from each hospital, predominantly female (n=343; 80%). The mean age of participants from hospitals A, B, and C was 36.81 (±7.03), 39.50 (±7.79), and 39.76 (±10.08) years, respectively. Tables 1 and 2 present participant characteristics.

**Table 1:** Personal and professional data of nursing professionals at three federal university hospitals, Brazil, 2022 (N = 429)

Variables	Hospital			Category		Total n (%)
	A n (%)	B n (%)	C n (%)	Nurses n (%)	Nursing technicians n (%)	
**Sex**						
Female	111 (77.6)	117 (81.8)	115 (80.4)	122 (84.1)	221 (77.8)	343 (80.0)
Male	32 (22.4)	26 (18.2)	28 (19.6)	23 (15.9)	63 (22.2)	86 (20.0)
**Position**						
Nurse	50 (35.0)	43 (30.1)	52 (36.4)	-	-	145 (33.8)
Nursing technician	93 (65.0)	100 (69.9)	91 (63.6)	-	-	284 (66.2)
**Type of unit**						
Inpatient/outpatient	111 (77.6)	98 (68.5)	119 (83.2)	106 (73.1)	222 (78.2)	328 (76.5)
Intensive care	32 (22.4)	45 (31.5)	24 (16.8)	39 (26.9)	62 (21.8)	101 (23.5)
**Higher level of education**						
High school	45 (31.5)	34 (23.8)	48 (33.6)	-	127 (44.7)	127 (29.6)
Higher education	38 (26.6)	37 (25.9)	35 (24.5)	12 (8.2)	98 (34.5)	110 (25.6)
Graduate education	60 (41.9)	72 (50.3)	60 (41.9)	133 (91.8)	59 (20.8)	192 (44.8)
**Work period**						
Day shift	87 (60.8)	81 (56.6)	92 (64.3)	86 (59.3)	174 (61.2)	260 (60.6)
Night shift	56 (39.2)	51 (35.7)	48 (33.6)	56 (38.6)	99 (34.8)	155 (36.1)
Rotation between shifts	0 (0.0)	11 (7.7)	3 (2.1)	3 (2.1)	11 (4.0)	14 (3.3)
**Intention to leave the position/role**						
No	138 (96.5)	131 (91.6)	130 (90.9)	137 (94.5)	262 (92.2)	399 (93.0)
Yes	5 (3.5)	12 (8.4)	13 (9.1)	8 (5.5)	22 (7.8)	30 (7.0)
**Frequency of staff inadequacy**						
≥50% of the time worked	57 (40.0)	103 (72.0)	91 (63.7)	80 (55.0)	171 (60.2)	251 (58.5)
< 50% of the time worked	86 (60.0)	40 (28.0)	52 (36.3)	65 (45.0)	113 (39.8)	178 (41.5)
**Job satisfaction/satisfaction with the current position/role**						
Satisfied	127 (88.8)	92 (64.3)	105 (73.4)	110 (75.8)	214 (75.3)	324 (75.5)
Dissatisfied	16 (11.2)	51 (35.7)	38 (26.6)	35 (24.2)	70 (24.7)	105 (24.5)

**Table 2:** Age, current job experience, current department/unit experience, and number of patients on current shift at three federal university hospitals, Brazil, 2022 (N = 429)

Variable	Minimum	Mean	Standard deviation	Q1	Median	Q3	Maximum
Age (years)	23	38.90	7.55	33	38	43	61
Years of experience in current position (years)	0	12.10	6.70	7	10	16	40
Years of experience in the unit (years)	0	4.55	4.88	1	3	6	37
Number of patients on current shift	0	7.37	7.91	3	5	9	100

*Q - quartile.*

There was no association between the variables “intention to leave nursing” and “hospital” (*p*=0.130). Therefore, it was decided to conduct the following analyses using data from hospitals A, B, and C in combination. Thus, an association was found between intention to leave work and personal and work-related variables of nursing professionals, with a higher intention among professionals who had higher education or graduate education (*p*=0.029), who were not satisfied with their job (*p*<0.001), who considered the number of professionals on the staff inadequate to provide care (*p*=0.026), younger professionals (*p*=0.014) and with less experience in the position (*p*=0.046) (Table 3).

**Table 3:** Association between intention to leave work and variables of personal and work characteristics and job satisfaction among nursing professionals at three federal university hospitals, Brazil, 2022 (N = 429)

Variables		Intention to leave the position		*p* value^*^
		Não n (%)	Sim n (%)	
**Sex**	Female	323 (94.2)	20 (5.8)	0.055**^1^**
	Male	76 (88.4)	10 (11.6)	
**Position**	Nursing technician	262 (92.3)	22 (7.7)	0.260**^1^**
	Nurse	137 (94.5)	8 (5.5)	
**Unit type**	Inpatient/outpatient care	303 (92.4)	25 (7.6)	0.249^1^
	Intensive care	96 (95.0)	5 (5.0)	
Shift^**^	Day shift	243 (93.5)	17 (6.5)	0.390^1^
	Night shift	143 (92.3)	12 (7.7)	
**Educational level**	Médio	123 (96.9)	4 (3.1)	0.029^1*^
	High school or graduate education	276 (91.4)	26 (8.6)	
**Job satisfaction/satisfaction with the current position/role**	Yes	310 (95.7)	14 (4.3)	<0.001^1*^
	No	89 (84.8)	16 (15.2)	
**Frequency of staff inadequacy**	≥50% of the time	228 (90.8)	23 (9.2)	0.026^1*^
	>50% of the time	171 (96.1)	7 (3.9)	
		**Mean (Q_1_-Q_3_)**		
**Age (years)**		38.0 (34.0-43.0)	35.5 (30.8-39.5)	0.014^2*^
**Years of experience in current role**		11.0 (7.0-16.0)	10.0 (6.0-12.5)	0.046^2*^
**Years of experience in the unit**		3.0 (1.0-6.0)	2.0 (1.0-6.0)	0.342**^2^**
**Number of patients on current shift**	5.0 (3.0-10.0)	5.0 (3.0-7.3)	0.919**^2^**

^1^Chi-square test; ^2^Mann-Whitney test; ^*^Statistically significant p<0.05; ^**^n=415 (14 participants who work rotating shifts were excluded; Q - quartile.

To assess variables associated with intention to leave work in combination with variables of personal and work characteristics and job satisfaction of nurses and nursing technicians, a logistic regression analysis was performed. In the multivariate analysis, variables with p < 0.05 in the univariate analysis were included; however, only job satisfaction showed an association with intention to leave work (OR=3.519; CI=1.594-7.766; *p*=0.002). This showed that job satisfaction was associated with intention to leave nursing, i.e., the lower the job satisfaction, the greater the chance of intending to leave the job, being three times higher (Table 4).

**Table 4:** Variables associated with intention to leave work among nurses and nursing technicians from three federal university hospitals, Brazil, 2022 (N = 429)

Variables	Odds Ratio	95%CI	*p* value
Age	0.975	0.907 - 1.047	0.486
Years of experience in current role	0.954	0.876 - 1.039	0.281
Educational level	0.368	0.123 - 1.105	0.075
Frequency of staff inadequacy	0.537	0.215 - 1.344	0.184
Job satisfaction	3.519	1.594 - 7.766	**0.002** ^*^

CI - Confidence Interval;

* Statistically significant p<0.05.

## DISCUSSION

This study sought to analyze factors associated with intention to leave work among nursing professionals at three university hospitals in Brazil, identifying that dissatisfied professionals are up to three times more likely to intend to leave their jobs when compared to those satisfied with their position.

The characterization of participants from the three hospitals surveyed shows a predominance of female respondents, under 40 years of age, and nursing technicians. These data are corroborated by a sociodemographic study on nursing professionals in Brazil, which found that: 85.1% of the nursing staff is composed of female professionals; 61.7% were under 40 years of age; and 77% of the professionals interviewed were a nursing technician or nursing assistant^(15)^.

A higher intention to leave their jobs was identified among professionals who had a higher education degree or graduate education (*p*=0.029) in this investigation. Regarding the highest educational level, it was found that 70.4% of the professionals interviewed had higher education and/or graduate education, and, among them, 33.8% are nurses. This shows that 36.6% of nursing technicians in the sample analyzed across the three hospitals have undergraduate or graduate education. A nationwide study showed that only 11.5% of nursing technicians in Brazil have completed higher education^(15)^. One possible explanation for the situation observed in the research settings could be the selection process through public competitive examinations/selections. In these modalities, many professionals enter as nursing technicians while undergoing higher education and, due to the differentiated remuneration compared to the job market, choose to remain in the position even after completing their university education. A context favorable to continuing education in teaching hospitals after joining the institution must also be considered.

In relation to intention to leave the position/role within one year, this study found prevalences of 3.5%, 8.4%, and 9.1%, respectively, in institutions A, B, and C, with a total percentage of 7.0%. This variation between hospitals can be explained by the timing of data collection, since in hospital A it occurred in the pre-pandemic period, while in hospitals B and C it occurred during the COVID-19 pandemic. There is evidence that the pandemic period influenced the nursing staff’s work process^(16)^. It was necessary to reorganize the entire work process, in addition to the emotional impact of caring for patients with a little-known, highly transmissible disease, and the risk of contamination and death. The high demand for care for complex patients with severe conditions led to an excessive workload in healthcare institutions, increasing intention to leave nursing and the profession^(17,18)^.

A study conducted in southern Brazil assessed intention to leave nursing during the COVID-19 pandemic in different settings and care levels, with a prevalence of 24.6% among participants^(17)^. From an international perspective, a Swiss study found a prevalence of 55% among nursing professionals who considered abandoning their careers during the COVID-19 pandemic^(18)^. A systematic review and meta-analysis study identified that approximately one-third of nursing professionals who worked during the COVID-19 pandemic indicated an intention to leave their jobs^(19)^. Outside the pandemic context, in the pre-pandemic period, a study conducted in 40 hospitals in Italy found that 35.5% of nurses wanted to leave nursing due to factors such as emotional exhaustion, high patient/nurse ratios, and lack of personal fulfillment^(20)^.

The lower prevalence of intention to leave work in this study can be explained by the type of employment contract - specifically, public competitive examinations/selection processes - in the participating institutions. This provides job stability to successful candidates and creates a qualified staff, possibly contributing to a sense of professional fulfillment. Furthermore, the remuneration offered in these institutions is higher than salaries found in the national market^(15)^, in addition to the prestige and professional development opportunities associated with working at a federal teaching hospital, these factors may explain the low intention to leave nursing in the research settings.

In this study, intention to leave work was associated with age, being more prevalent among younger workers (*p*=0.014) and with less experience in the position (*p*=0.046). Systematic review studies have identified age as one of the factors associated with intention to leave work, with younger workers having a higher intention to leave their jobs^(9,21)^. A study conducted in the United States with 207.636 nurses identified a 21% prevalence of intention to leave their jobs, with the likelihood of reporting such intention decreasing by 1.5% for each year of job experience. In the same study, professionals aged 22 to 37 reported a higher intention to leave their jobs compared to others, with the exception of those aged 73 to 90, due to their intention to retire^(22)^.

In this study, nursing professionals reported feeling that the staff was quantitatively inadequate for more than half of their working time and had a greater intention to leave their jobs (*p*=0,026). The work overload resulting from insufficient staff, material resources, high patient demand, and unit management and organization can affect the nursing staff mentally and physically, directly impacting patient safety^(23)^ and intention to leave nursing and the profession^(1,21)^. Nurses, as nursing staff leaders, must work with managers to achieve the best quantitative and qualitative nursing staff organization, considering the complexity and multidimensionality of the activities performed in the care and management contexts^(24,25)^, with a focus on quality of care and patient safety^(26)^.

The shortage of nursing professionals is a growing problem globally and could worsen in the coming years due to the high intention of leaving the profession. An additional concern in developing countries relates to the trend of nurses migrating to countries with better economic conditions, compromising the number of professionals available in more vulnerable areas^(27)^. Furthermore, talent loss, also known as brain drain, represents a growing trend in nursing with potential consequences related to the loss of qualified professionals and an increased workload for remaining professionals, while migrants may experience difficulties related to adapting to the destination country and may encounter xenophobia^(28)^.

In this study, reports of satisfaction with the current position predominated, as observed in another investigation^(29)^. This result differs from that observed in other studies, in which satisfaction was less prevalent among nursing professionals. Studies conducted during the COVID-19 pandemic identified high job satisfaction in 11.6% of nurses in Iran^(30)^ and in 25.2% of nurses in Egypt^(31)^. A Brazilian study identified satisfaction among 26.9% of participants, with a prevalence of ambivalent feelings regarding satisfaction (72.1%)^(32)^. Fear of the disease and high workloads have influenced job satisfaction among frontline COVID-19 nursing professionals in the Philippines, increasing their intention to leave their jobs^(33)^. It is worth mentioning that, in the concept of satisfaction, different criteria are perceived by each worker.

Despite this, low job satisfaction was associated with intention to leave nursing in this investigation (*p*<0.001). Intention to leave nursing was three times higher among professionals who reported dissatisfaction with their current position (OR=3.519; CI=1.594-7.766; *p*=0.002). Job dissatisfaction has been identified as one of the factors related to intention to leave a job or profession in various studies^(1,21,34)^.

Nursing professionals’ low availability represents a serious risk to achieving the SDGs^(27,35)^, not only in goal three, explicitly related to health, but in all the others, since nursing practice has a broad interface with the SDGs^(4)^. Strategies capable of improving the retention of nursing professionals include reducing workload, improving working conditions, investing in career plans and better remuneration, improving staff sizing, promoting nursing leadership, ensuring social support, and favoring work-life balance^(34)^, thereby promoting greater job satisfaction.

### Study limitations

This research has limitations, as it is situated within the context of a federal university hospital, where job security and remuneration are factors that can impact intention to leave work. Added to this are the limitations of the cross-sectional research design, which presents only a snapshot in time of the phenomenon studied. Therefore, replication of this study in other contexts and time periods, as well as at different levels of care, is recommended.

Furthermore, the COVID-19 pandemic was a landmark event that exposed the weaknesses of healthcare systems worldwide, reflecting the importance of organizing work in healthcare. It was a time of exhausting caregiving that directly impacted job satisfaction and intention to leave nursing, highlighting the need for further studies on the subject and the development of public policies to encourage and value nursing professionals.

### Contributions to nursing, health, or public policy

This study presents relevant data and sheds light on an emerging issue for Brazilian nursing, which may experience a shortage of professionals in a few years. In this regard, identifying nurses and nursing technicians who intend to leave their jobs and the profession signals to nursing leadership and health service managers the need to (re)examine the potential factors associated with this problem.

It is worth highlighting that the investigation carried out in three Brazilian hospitals from different regions of the country points to common determining variables, indicating the pressing need to discuss public policies for nursing personnel, such as investment in better working conditions, appreciation and recognition of nursing, continuing education, among others. It is believed that, in this direction, it will be possible to promote nursing workforce retention, considered one of the fundamental pillars for guaranteeing quality and safe healthcare for the population.

## CONCLUSIONS

This investigation indicated that job dissatisfaction can increase the likelihood of wanting to leave a job by up to three times. Among the study participants, intention to leave a job was associated with younger professionals, those with less experience in their role, those with higher education or graduate education, those dissatisfied with their jobs, and those who considered the number of nursing professionals inadequate for most of the working time. Although the percentage of people intending to leave their jobs in this study is lower than those identified in international studies, it highlights the growing concern about potential shortages of nursing professionals in Brazil and the migration of Brazilian nursing talent abroad. The need to expand national studies on this topic is highlighted, in order to identify intention to leave nursing within the national context, as well as the importance of investment to retain these professionals in the country.

## Data Availability

The research data are available only upon request.

## References

[1] Vries N, Boone A, Godderis L, Bouman J, Szemik S, Matranga D (2023). The race to retain healthcare workers: a systematic review on factors that impact retention of nurses and physicians in hospitals. Inquiry.

[2] World Health Organization (WHO) (2020). The State of the world’s nursing 2020: investing in education, jobs and leadership.

[3] Buchan J, Catton H. (2023). Recover to rebuild: Investing in the nursing workforce for health system effectiveness.

[4] Rosa WE, Catton H, Davidson PM, Hannaway CJ, Iro E, Klopper HC (2021). Nurses and Midwives as Global Partners to Achieve the Sustainable Development Goals in the Anthropocene. J Nurs Scholarsh.

[5] Instituto Brasileiro de Geografia e Estatística (IBGE) (2024). Indicadores Brasileiros para os Objetivos de Desenvolvimento Sustentável.

[6] Organização Pan-Americana da Saúde (OPAS) (2019). Diretriz Estratégica para a enfermagem na Região das Américas.

[7] Mendes M, Martins MS, Acordi I, Ramos FRS, Brehmer LCF, Pires DEP. (2022). Nursing workforce: scenario and trends. Rev Enferm UFSM.

[8] Stafford S, Avsar P, Nugent L, O’Connor T, Moore Z, Patton D (2022). What is the impact of patient violence in the emergency department on emergency nurses’ intention to leave?. J Nurs Manag.

[9] Al Yahyaei A, Hewison A, Efstathiou N, Carrick-Sen D. (2022). Nurses’ intention to stay in the work environment in acute healthcare: a systematic review. J Res Nurs.

[10] Pressley C, Newton D, Garside J, Simkhada P, Simkhada B. (2022). Global migration and factors that support acculturation and retention of international nurses: a systematic review. Int J Nurs Stud Adv.

[11] Silva JF, Rodrigues MJ, Silva MR. (2022). Utilização do kobotoolbox para caracterização das unidades de saúde da rede municipal de Jataí-GO, 2019. Hygeia.

[12] Cohen J. (2013). Statistical Power Analysis for the Behavioral Sciences.

[13] Kalisch BJ, Landstrom G, Williams RA. (2009). Missed nursing care: errors of omission. Nurs Outlook.

[14] Siqueira LDC, Caliri MHL, Haas VJ, Kalisch B, Dantas RAS. (2017). Validation of the MISSCARE-BRASIL survey: a tool to assess missed nursing care. Rev Latino-Am Enfermagem.

[15] Silva MCN, Machado MH. (2020). Sistema de saúde e trabalho: desafios para a Enfermagem no Brasil. Cien Saude Colet.

[16] Usberg G, Clari M, Conti A, Põld M, Kalda R, Kangasniemi M. (2024). Changes in nurses’ work: a comparative study during the waves of COVID-19 pandemic. Int J Nurs Pract.

[17] Kantorski LP, Oliveira MM, Alves PF, Treichel CAS, Wünsch CG, Santos LH (2022). Intention to leave Nursing during the COVID-19 pandemic. Rev Lat Am Enfermagem.

[18] Andersson M, Fredholm A, Nordin A, Engström Å. (2023). Moral distress, health and intention to leave: critical care nurses’ perceptions during COVID-19 pandemic. SAGE Open Nurs.

[19] Ulupınar F, Erden Y. (2024). Intention to leave among nurses during the COVID-19 outbreak: a rapid systematic review and meta-analysis. J Clin Nurs.

[20] Sasso L, Bagnasco A, Catania G, Zanini M, Aleo G, Watson R. (2019). Push and pull factors of nurses’ intention to leave. J Nurs Manag.

[21] Vries N, Maniscalco L, Matranga D, Bouman J, Peter de Winter J (2024). Determinants of intention to leave among nurses and physicians in a hospital setting during the COVID-19 pandemic: a systematic review and meta-analysis. PLoS One.

[22] Koehler T, Olds D. (2022). Generational differences in nurses’ intention to leave. West J Nurs Res.

[23] Silva FX, Santos MA, Queiroz SS, Oliveira TF, Ferreira FCL, Cavalcanti EO. (2023). Nursing team overload and the risk of adverse events. Nursing.

[24] Guardalupe JA, Brum ID, Canto DF, Telles KCM, Magalhães AMM, Oliveira JLC. (2023). Comparison of patient classification systems for dimensioning nursing staff. Rev Esc Enferm USP.

[25] Moraes RMR, Nishiyama JAP, Báo ACP, Costa FM, Aldabe LN, Oliveira JLC. (2021). Sizing of nursing staff in clinical, surgical and pediatric hospitalization units. Texto Contexto Enferm.

[26] Trovó SA, Cucolo DF, Perroca MG. (2020). Time and quality of admissions: nursing workload. Rev Bras Enferm.

[27] Fields L, Perkiss S, Dean BA, Moroney T. (2021). Nursing and the sustainable development goals: a scoping review. J Nurs Scholarsh.

[28] Ulupinar S, Şen Y, Eycan Ö. (2024). Nurses’ attitudes toward brain drain and the associated factors. Am J Nurs.

[29] Santos LA, Uzeda AL, Garcia LR, Goulart MCL, Góes FGB, Santos JL. (2023). Nursing work during the COVID-19 pandemic: (dis)satisfaction and (de)motivation. Rev Rene.

[30] Heidari S, Parizad N, Goli R, Mam-Qaderi M, Hassanpour A. (2022). Job satisfaction and its relationship with burnout among nurses working in COVID-19 wards: a descriptive correlational study. Ann Med Surg.

[31] Said RM, El-Shafei DA. (2021). Occupational stress, job satisfaction, and intent to leave: nurses working on front lines during COVID-19 pandemic in Zagazig City, Egypt. Environ Sci Pollut Res Int.

[32] Vilas M, Pirino B, Lopes C, Sobrinho N, Dini AP. (2023). Professional satisfaction in nursing during the COVID-19 pandemic. Rev Latino-Am Enfermagem.

[33] Labrague LJ, Santos JAA. (2021). Fear of COVID-19, psychological distress, work satisfaction and turnover intention among frontline nurses. J Nurs Manag.

[34] Tamata AT, Mohammadnezhad M. (2023). A systematic review study on the factors affecting shortage of nursing workforce in the hospitals. Nurs Open.

[35] Fields L, Dean BA, Perkiss S, Moroney T. (2024). Nursing action towards the sustainable development goals: barriers and opportunities. Nurse Educ Today.

