# Supplementary material for: Intention to leave work among nursing professionals at three university hospitals in Brazil
Source: Rev Bras Enferm. 2026 Jul 6;79:e20250192. doi: 10.1590/0034-7167-2025-0192 (PMC13336233; doi:10.1590/0034-7167-2025-0192)
Supplement: Translation

# Translation: Intenção de deixar o emprego entre profissionais de enfermagem de três hospitais universitários do Brasil

**DOI:** 10.1590/0034-7167-2025-0192pt

**Authors:** Herica Silva Dutra, Diego Marques de Oliveira, Adrize Rutz Porto, Maria Cristina Soares Rodrigues, Diana Albuquerque Alvim de Paula, Paula Roberta Silva Araújo Machado, Lucas da Silva Dellalibera, Nádia Fontoura Sanhudo

**Affiliations:** I Universidade Federal de Juiz de Fora. Juiz de Fora, Minas Gerais, Brasil; II Universidade Federal de Pelotas. Pelotas, Rio Grande do Sul, Brasil; III Universidade de Brasília. Brasília, Distrito Federal, Brasil

**Keywords:** Enfermagem, Intenção, Emprego, Reorganização de Recursos Humanos, Satisfação no Emprego.

## Abstract

**Objetivos:**

analisar fatores associados à intenção de deixar o emprego entre profissionais de enfermagem em três hospitais universitários federais do Brasil.

**Métodos:**

estudo multicêntrico transversal. Coletaram-se dados entre outubro/2018 e setembro/2022, incluindo dados pessoais e laborais, satisfação com o cargo, e intenção de deixar o emprego. Foram utilizados os testes qui-quadrado, Mann-Whitney e regressão logística.

**Resultados:**

participaram 429 profissionais de enfermagem com idade média de 38,9 anos, predominantemente do sexo feminino (80%), técnicos de enfermagem (66,2%). Apresentaram maior intenção de deixar o emprego aqueles com formação em nível superior ou pós-graduação (*p*=0,029), insatisfeitos com o emprego (*p*<0,001), que consideravam quantitativo da equipe inadequado (*p*=0,026), mais jovens (*p*=0,014) e com menor tempo de experiência (*p*=0,046). A baixa satisfação com o cargo foi associada com três vezes maior chance de deixar o emprego (OR=3,519; IC=1,594-7,766; *p*=0,002).

**Conclusões:**

profissionais de enfermagem insatisfeitos têm maior chance de intenção de deixar o emprego.

## INTRODUÇÃO

A qualidade do cuidado com o usuário pode ser refletida diretamente pela capacidade da instituição de saúde em reter seus trabalhadores, especialmente os profissionais de enfermagem, que estão a maior parte do tempo em contato com o paciente^(1)^. Mundialmente, a escassez de profissionais de enfermagem foi estimada em 6 milhões de profissionais no período pré-pandêmico, com perspectivas de chegar a 13 milhões nos próximos anos como consequência do aumento da demanda de cuidados e da falta de políticas de retenção desses profissionais^(2)^. O Conselho Internacional de Enfermagem, porém, destaca números mais alarmantes, estimando um déficit de até 30,6 milhões de profissionais de enfermagem globalmente^(3)^.

Considerando esse cenário e tendências, a Organização das Nações Unidas traça os Objetivos de Desenvolvimento Sustentável (ODS), cujo número três visa “garantir o acesso à saúde de qualidade e promover o bem-estar para todos, em todas as idades”^(4)^. Para que isso ocorra, será necessário “aumentar substancialmente o financiamento da saúde e o recrutamento, desenvolvimento e formação, e retenção do pessoal de saúde nos países em desenvolvimento”^(5)^. Destaca-se que os profissionais de enfermagem representam a maior força de trabalho em saúde. Sendo assim, alcançar saúde para todos está diretamente relacionada à capacidade da enfermagem em atingir seu potencial de contribuição com qualidade na saúde global. Isso pode ser feito por meio da aplicação de seus conhecimentos, valores e competências práticas, permitindo responder às demandas atuais e futuras de saúde^(3)^.

Nas Américas, devido às desigualdades existentes entre os países, vários fatores têm contribuído para a falta de enfermeiros e técnicos de enfermagem, tais como migrações e mobilidades de mão de obra, má distribuição dos profissionais de saúde, valorização e reconhecimento profissional baixos, deficiências na formação profissional^(6)^, entre outros. Caminhando na contramão à tendência mundial, o Brasil conta com 70% da força de trabalho em saúde representada pela enfermagem, com perspectiva de aumento em 51% até 2030. Contudo, a desigual distribuição profissional em diferentes localidades, aliada à falta de políticas de retenção dos trabalhadores, tem contribuído para aumento da insatisfação com a profissão e a intenção de deixá-la^(7)^.

Intenção de deixar o emprego pode ser entendida como resultado de um longo processo de recorrentes exposições a fatores estressantes no ambiente do trabalho, aumentando insatisfação com o emprego, distanciamento emocional da função e sentimento de deixar o trabalho como formas de lidar com o agente estressor^(8-10)^. Entre os fatores que influenciam a intenção de deixar o emprego, no contexto hospitalar, há excessiva carga de trabalho, inadequação quantitativa e qualitativa da equipe de enfermagem, exaustão emocional, conflito interprofissional, demanda por atividades com esforço físico, e violência no ambiente de trabalho. O ambiente da prática de enfermagem pode adicionalmente ser desfavorável, considerando estilos de gerência e liderança do enfermeiro, falta de empoderamento dos profissionais de enfermagem, falta de suporte, clima organizacional ruim, ausência de planos de carreira, insatisfação no emprego, e pouco reconhecimento social e profissional^(8,9)^.

Apesar de a intenção em deixar o trabalho ser um fenômeno já bem documentado e preocupante nos países desenvolvidos^(1)^, a contribuição de estudos brasileiros sobre é temática é pequena quando comparada a outros países, conforme identificado em extensa revisão sistemática que avaliou 345 estudos sobre a prevalência e fatores determinantes para retenção de profissionais de enfermagem^(1)^. Além disso, não foram identificados estudos multicêntricos brasileiros prévios discutindo a intenção de deixar o emprego e a profissão. Desataca-se ainda que, no Brasil, a perda de profissionais qualificados para o mercado internacional, somada à insatisfação com a profissão, pode contribuir para o aumento da vulnerabilidade do sistema de saúde brasileiro^(6)^. Tais aspectos, somados às projeções de déficit de profissionais de enfermagem para os próximos anos globalmente, justificam a relevância desta investigação.

## OBJETIVOS

Analisar fatores associados à intenção em deixar o emprego entre profissionais de enfermagem em três hospitais universitários do Brasil.

## MÉTODOS

### Aspectos éticos

Esta pesquisa foi submetida à apreciação por Comitês de Ética em Pesquisa das três instituições participantes. Foram respeitados os preceitos da Resolução no 466/12 do Conselho Nacional de Ética. Os dados foram obtidos após o consentimento dos participantes, por meio de concordância expressa no Termo de Consentimento Livre e Esclarecido (TCLE).

### Desenho, período e local do estudo

Trata-se de estudo multicêntrico, transversal, com abordagem quantitativa, descrito no presente artigo em conformidade com o *STrengthening the Reporting of OBservational studies in Epidemiology* (STROBE). O estudo envolveu três hospitais universitários federais caracterizados como hospital geral de médio porte, geridos pela Empresa Brasileira de Serviços Hospitalares (Ebserh). Os hospitais participantes do estudo estão localizados em três regiões brasileiras, a saber: Centro-Oeste, Sudeste, e Sul. A fim de preservar a identificação dos cenários, as instituições serão denominadas como hospitais A, B e C.

A coleta de dados ocorreu em momentos e formatos distintos nos três cenários, devido às restrições impostas pela pandemia de COVID-19. Além disso, a apreciação ética nos cenários envolvidos ocorreu em momentos distintos, devido a variações nos trâmites documentais e burocráticos, e estabelecimento de prioridade para estudos relacionados à COVID-19 durante a pandemia.

No hospital A, os dados foram coletados entre outubro de 2018 e abril de 2019. O convite foi feito pessoalmente pelos pesquisadores, assim como a entrega e assinatura do TCLE impresso. O formulário da pesquisa foi respondido em dispositivo eletrônico *Android* cedido pelos pesquisadores.

No hospital B, a coleta de dados aconteceu entre novembro de 2020 e junho de 2021. O convite foi feito pessoalmente, e o TCLE impresso entregue aos participantes. O formulário da pesquisa foi disponibilizado por meio de *link* eletrônico via aplicativo de mensagem (*WhatsApp^®^*), e as respostas foram preenchidas nos dispositivos dos próprios participantes.

No hospital C, os dados foram colhidos entre julho de 2021 e setembro de 2022. O TCLE e o *link* do formulário da pesquisa foram enviados aos participantes por e-mail institucional. Devido à baixa adesão nessa modalidade, após modificação do cenário epidemiológico, foi autorizada a abordagem presencial dos participantes para coleta de dados. Assim, o TCLE e o *link* do formulário foram disponibilizados diretamente ao participante por meio de aplicativo de mensagem (*WhatsApp^®^*), o que facilitou a obtenção das respostas.

O formulário de pesquisa eletrônico foi disponibilizado por meio do *software KoBoToolbox* ^(11)^. Trata-se de ferramenta de acesso gratuito para coleta de dados que pode ser utilizada em formato remoto ou presencial, com ou sem suporte de internet.

Ressalta-se que, apesar da abordagem distinta de convite e acesso ao formulário eletrônico com o instrumento de coleta de dados, todos os participantes responderam ao mesmo formulário padronizado e disponibilizado por meio do *software KoBoToolbox*. Todos os participantes tiveram a oportunidade de esclarecer suas dúvidas a respeito do preenchimento do formulário, e não tiveram qualquer limitação de tempo para completar as respostas.

### População ou amostra; critérios de inclusão e exclusão

O número total de 429 participantes foi calculado considerando o poder do teste estatístico^(12)^, com auxílio do software G*Power 3.1.9.3. Para delimitação do tamanho amostral, foram adotados o tamanho do efeito médio (*effect size*) e as possibilidades de erros tipo I (α = 0,05) e II (β = 0,80). Assim, obteve-se uma amostra de 143 participantes em cada cenário considerando o teste qui-quadrado de Pearson.

Os participantes atenderam aos seguintes critérios de inclusão no estudo: a) ser enfermeiro ou técnico de enfermagem; b) possuir vínculo empregatício há no mínimo três meses com a instituição alvo de pesquisa (devido ao período de experiência ser de três meses); c) não estar de férias ou licença no momento da coleta de dados. O critério de exclusão foi: a) desenvolver atividades exclusivamente de cunho administrativo ou gerencial (devido ao interesse na perspectiva da equipe assistencial). Nos hospitais A e C, foi adotada amostra aleatória com reposição estratificada para enfermeiros e técnicos de enfermagem. O sorteio foi realizado a partir de lista fornecida pela direção de enfermagem. No hospital B, foi adotada amostra por conveniência.

### Protocolo do estudo

Para este estudo, foram adaptadas as variáveis que constam em “informação geral” do instrumento MISSCARE-BRASIL. O MISSCARE foi desenvolvido em língua inglesa^(13)^ e, posteriormente, validado no Brasil, com a inclusão de itens que refletissem melhor a realidade brasileira^(14)^. Neste estudo, foram consideradas apenas as variáveis de caracterização pessoal e laboral, além da satisfação no trabalho e intenção de deixar o emprego.

As variáveis de caracterização incluíram sexo (masculino/feminino), idade (em anos), cargo (enfermeiro/técnico de enfermagem), nível educacional mais elevado (ensino médio/ensino superior/pós-graduação), tempo de experiência no cargo (em anos), tempo de experiência na unidade (em anos), período de trabalho (diurno/noturno/rodízio entre os turnos), número de pacientes atendidos no plantão atual e frequência que sente se inadequado o número de funcionários da sua unidade/setor (0%, 25%, 50%, 75% e 100% do tempo).

A intenção em deixar a profissão foi avaliada por meio da pergunta “Planeja deixar cargo ou função atual?”, com alternativas de resposta “nos próximos seis meses”, “no próximo ano” e “não planejo deixar meu cargo/função atual”. Em seguida, a satisfação profissional foi quantificada por meio da pergunta “o quanto você está satisfeito em seu cargo/função atual?”, com alternativas de resposta “muito satisfeito”, “satisfeito”, “nem satisfeito nem insatisfeito”, “insatisfeito” ou “muito insatisfeito”.

### Análise dos resultados e estatística

Os dados foram tabulados no programa *Microsoft Excel for Windows* ^®^. Foram elaboradas tabelas de frequência (absoluta e percentual) para descrever a caracterização da amostra. Foram obtidas medidas de posição das variáveis contínuas (média, mediana, desvio padrão, quartis, valor máximo e valor mínimo).

Para fins de análise dos dados, foram agrupadas as respostas relativas à satisfação no trabalho da seguinte forma: as respostas “muito satisfeito” e “satisfeito” foram categorizadas como “satisfeito”; e as respostas “nem satisfeito nem insatisfeito”, “muito insatisfeito” e “insatisfeito” foram categorizadas como “insatisfeito”. Da mesma forma, para a variável “intenção de deixar o emprego”, as respostas “nos próximos seis meses” e “no próximo ano” foram agrupadas em “sim”. A variável “frequência que sente ser inadequado o número de funcionários da sua unidade/setor” foi agrupada em “maior ou igual que 50% do tempo” e “menor que 50% do tempo”.

A intenção de deixar o emprego foi a variável dependente, e as demais foram variáveis independentes. A fim de minimizar possíveis vieses, os seguintes procedimentos foram adotados: disponibilidade para esclarecimento de dúvidas aos participantes ao longo do desenvolvimento do estudo; garantia de anonimato na divulgação dos resultados; sem limitação de tempo para preenchimento do formulário de coleta de dados; e análise criteriosa e imparcial dos dados.

A associação entre as variáveis categóricas foi verificada por meio do teste qui-quadrado de Pearson. A relação entre as variáveis contínuas e a intenção de deixar o emprego foi verificada por meio do teste de Mann-Whitney, considerando o resultado do teste de normalidade das variáveis quantitativas. Para avaliar a associação das variáveis independentes com a variável dependente “intenção de deixar o emprego” dicotomizada (sim/não), realizou-se a regressão logística. Utilizou-se o método *Enter*, e para verificação do ajuste do modelo final, foi considerado o teste de Hosmer & Lemeshow (*X* ^2^=9,562; p=0,297). As análises estatísticas foram realizadas com apoio do *software Statistical Package for the Social Sciences*, versão 30. Foi considerado valor de p <0,05 para os testes estatísticos empregados.

## RESULTADOS

Participaram 429 profissionais de enfermagem, sendo 143 em cada hospital, predominantemente do sexo feminino (n=343; 80%). A média de idade dos participantes dos hospitais A, B e C foi de 36,81 (±7,03), 39,50 (±7,79) e 39,76 (±10,08) anos, respectivamente. A caracterização dos participantes é apresentada nas Tabelas 1 e 2.

**Tabela 1:** Dados pessoais e laborais dos profissionais de enfermagem em três hospitais universitários federais, Brasil, 2022 (N = 429)

Variáveis	Hospital			Categoria		Total n (%)
	A n (%)	B n (%)	C n (%)	Enfermeiros n (%)	Técnicos de enfermagem n (%)	
**Sexo**						
Feminino	111 (77,6)	117 (81,8)	115 (80,4)	122 (84,1)	221 (77,8)	343 (80,0)
Masculino	32 (22,4)	26 (18,2)	28 (19,6)	23 (15,9)	63 (22,2)	86 (20,0)
**Cargo**						
Enfermeiro	50 (35,0)	43 (30,1)	52 (36,4)	-	-	145 (33,8)
Técnico de enfermagem	93 (65,0)	100 (69,9)	91 (63,6)	-	-	284 (66,2)
**Tipo de unidade**						
Internação/ambulatório	111 (77,6)	98 (68,5)	119 (83,2)	106 (73,1)	222 (78,2)	328 (76,5)
Terapia intensiva	32 (22,4)	45 (31,5)	24 (16,8)	39 (26,9)	62 (21,8)	101 (23,5)
**Nível educacional mais elevado**						
Ensino médio	45 (31,5)	34 (23,8)	48 (33,6)	-	127 (44,7)	127 (29,6)
Ensino superior	38 (26,6)	37 (25,9)	35 (24,5)	12 (8,2)	98 (34,5)	110 (25,6)
Pós-graduação	60 (41,9)	72 (50,3)	60 (41,9)	133 (91,8)	59 (20,8)	192 (44,8)
**Período de trabalho**						
Diurno	87 (60,8)	81 (56,6)	92 (64,3)	86 (59,3)	174 (61,2)	260 (60,6)
Noturno	56 (39,2)	51 (35,7)	48 (33,6)	56 (38,6)	99 (34,8)	155 (36,1)
Rodízio entre os turnos	0 (0,0)	11 (7,7)	3 (2,1)	3 (2,1)	11 (4,0)	14 (3,3)
**Intenção de deixar o cargo/função**						
Não	138 (96,5)	131 (91,6)	130 (90,9)	137 (94,5)	262 (92,2)	399 (93,0)
Sim	5 (3,5)	12 (8,4)	13 (9,1)	8 (5,5)	22 (7,8)	30 (7,0)
**Frequência de inadequação da equipe**						
≥50% do tempo trabalhado	57 (40,0)	103 (72,0)	91 (63,7)	80 (55,0)	171 (60,2)	251 (58,5)
< 50% do tempo trabalhado	86 (60,0)	40 (28,0)	52 (36,3)	65 (45,0)	113 (39,8)	178 (41,5)
**Satisfação com o cargo/função atual**						
Satisfeito	127 (88,8)	92 (64,3)	105 (73,4)	110 (75,8)	214 (75,3)	324 (75,5)
Insatisfeito	16 (11,2)	51 (35,7)	38 (26,6)	35 (24,2)	70 (24,7)	105 (24,5)

**Tabela 2:** Idade, tempo de experiência no cargo atual, tempo de experiência no setor/unidade e número de pacientes no plantão atual em três hospitais universitários federais, Brasil, 2022 (N = 429)

Variável	Mínimo	Média	Desvio padrão	Q1	Mediana	Q3	Máximo
Idade (anos)	23	38,90	7,55	33	38	43	61
Tempo de experiência no cargo atual (anos)	0	12,10	6,70	7	10	16	40
Tempo de experiência na unidade (anos)	0	4,55	4,88	1	3	6	37
Número de pacientes no plantão atual	0	7,37	7,91	3	5	9	100

*Q - quartil.*

Não houve associação entre as variáveis “intenção de deixar o emprego” e “hospital” (*p*=0,130). Dessa forma, optou-se por conduzir as análises seguintes utilizando os dados dos hospitais A, B e C de forma combinada. Assim, foi verificada associação entre intenção de deixar o emprego e as variáveis pessoais e laborais dos profissionais de enfermagem, apresentando maior intenção os profissionais que tinham formação em nível superior ou pós-graduação (*p*=0,029), que não estavam satisfeitos com o emprego (*p*<0,001), que consideravam o número de profissionais na equipe inadequado para realizar a assistência (*p*=0,026), os profissionais mais jovens (*p*=0,014) e com menor tempo de experiência no cargo (*p*=0,046) (Tabela 3).

**Tabela 3:** Associação entre intenção de deixar o emprego e as variáveis de caracterização pessoal e laboral e satisfação com o cargo entre profissionais de enfermagem em três hospitais universitários federais, Brasil, 2022 (N = 429)

Variáveis		Intenção de deixar o cargo		Valor de *p* ^*^
		Não n (%)	Sim n (%)	
**Sexo**	Feminino	323 (94,2)	20 (5,8)	0,055**^1^**
	Masculino	76 (88,4)	10 (11,6)	
**Cargo**	Técnico em enfermagem	262 (92,3)	22 (7,7)	0,260**^1^**
	Enfermeiro	137 (94,5)	8 (5,5)	
**Tipo de unidade**	Internação/ambulatório	303 (92,4)	25 (7,6)	0,249^1^
	Terapia intensiva	96 (95,0)	5 (5,0)	
Turno^**^	Diurno	243 (93,5)	17 (6,5)	0,390^1^
	Noturno	143 (92,3)	12 (7,7)	
**Nível educacional**	Médio	123 (96,9)	4 (3,1)	0,029^1*^
	Superior ou pós-graduação	276 (91,4)	26 (8,6)	
**Satisfação com o cargo/função atual**	Sim	310 (95,7)	14 (4,3)	<0,001^1*^
	Não	89 (84,8)	16 (15,2)	
**Frequência de inadequação da equipe**	≥50% do tempo	228 (90,8)	23 (9,2)	0,026^1*^
	>50% do tempo	171 (96,1)	7 (3,9)	
		**Média (Q_1_-Q_3_)**		
**Idade (anos)**		38,0 (34,0-43,0)	35,5 (30,8-39,5)	0,014^2*^
**Tempo de experiência no cargo atual (anos)**		11,0 (7,0-16,0)	10,0 (6,0-12,5)	0,046^2*^
**Tempo de experiência na unidade (anos)**		3,0 (1,0-6,0)	2,0 (1,0-6,0)	0,342**^2^**
**Número de pacientes no plantão atual**	5,0 (3,0-10,0)	5,0 (3,0-7,3)	0,919**^2^**

1Teste qui-quadrado; 2Teste de Mann-Whitney; ^*^Estatisticamente significativo p<0,05; ^**^n=415 (foram excluídos 14 participantes que trabalham em rodízio entre turnos); Q - quartil.

Para avaliar as variáveis associadas com a intenção de deixar o emprego de maneira combinada com as variáveis de caracterização pessoal e laboral e satisfação com o cargo dos enfermeiros e técnicos de enfermagem, foi realizada análise de regressão logística. Na análise multivariada, foram inseridas as variáveis que tiveram *p* < 0,05 na análise univariada, porém apenas a satisfação com o cargo demonstrou associação com a intenção de deixar o emprego (OR=3,519; IC=1,594-7,766; *p*=0,002). Com isto, verificou-se que a satisfação com o cargo apresentou associação com a intenção de deixar o emprego, ou seja, quanto menor a satisfação com o cargo, maior chance intenção de deixar o emprego, sendo três vezes maior (Tabela 4).

**Tabela 4:** Variáveis associadas à intenção de deixar o emprego entre enfermeiros e técnicos de enfermagem de três hospitais universitários federais, Brasil, 2022 (N = 429)

Variáveis	*Odds Ratio*	IC95%	Valor de *p*
Idade	0,975	0,907 - 1,047	0,486
Tempo de experiência no cargo atual (anos)	0,954	0,876 - 1,039	0,281
Nível educacional	0,368	0,123 - 1,105	0,075
Frequência de inadequação da equipe	0,537	0,215 - 1,344	0,184
Satisfação com o cargo	3,519	1,594 - 7,766	**0,002** ^*^

IC - Intervalo de Confiança;

* Estatisticamente significativo p<0,05.

## DISCUSSÃO

Este estudo buscou analisar os fatores associados à intenção em deixar o emprego entre profissionais de enfermagem em três hospitais universitários do Brasil, identificando que os profissionais insatisfeitos têm até três vezes maior chance de intenção de deixar o emprego, quando comparados aos satisfeitos com o cargo.

A caracterização dos participantes dos três hospitais pesquisados mostra a predominância de respondentes mulheres, com idade inferior a 40 anos, no cargo de técnico de enfermagem. Esses dados são corroborados em estudo sociodemográfico sobre os profissionais de enfermagem no Brasil que encontrou que: 85,1% da equipe de enfermagem é composta por profissionais do sexo feminino; 61,7% tinham menos que 40 anos de idade; e 77% dos profissionais entrevistados exerciam a categoria profissional de técnico e auxiliar de enfermagem^(15)^.

Identificou-se maior intenção de deixar o emprego entre os profissionais que tinham formação em nível superior ou pós-graduação (*p*=0,029) nesta investigação. Com relação ao nível educacional mais elevado, foi constatado que 70,4% dos profissionais entrevistados possuíam ensino superior e/ou pós-graduação, e, entre eles, 33,8% são enfermeiros. Isto mostra que 36,6% dos técnicos de enfermagem da amostra analisada nos três hospitais possuem graduação ou pós-graduação. Estudo de abrangência nacional mostrou que apenas 11,5% dos técnicos de enfermagem do Brasil possuem ensino superior completo^(15)^. Uma possível explicação à situação observada nos cenários da pesquisa pode ser o processo de seleção por meio de concurso/seleção pública. Nessas modalidades, muitos profissionais ingressam como técnico de enfermagem em processo de formação em nível superior e, devido à remuneração diferenciada em relação ao mercado de trabalho, optam por permanecer no cargo, mesmo quando concluem a formação universitária. Há que se considerar também um contexto favorável à formação continuada nos hospitais de ensino após o ingresso na instituição.

Relativo à intenção de deixar o cargo/função em até um ano, este estudo encontrou nas instituições A, B e C uma prevalência de 3,5%, 8,4% e 9,1%, respectivamente, com percentual total de 7,0%. Essa variação entre os hospitais pode ser explicada devido ao momento em que a coleta de dados foi realizada, uma vez que, no hospital A, a mesma ocorreu no período pré-pandêmico, enquanto que, nos hospitais B e C, ocorreu no curso da pandemia de COVID-19. Há evidências de que o período de pandemia influenciou o processo de trabalho da equipe de enfermagem^(16)^. Foi necessário reorganizar todo o processo laboral, além do impacto emocional diante do cuidado a pacientes com uma doença pouco conhecida, com alta transmissibilidade, e do risco de contaminação e morte. A elevada demanda por atendimento aos pacientes complexos, com quadros graves, levou à excessiva carga de trabalho nas instituições de saúde, aumentando a intenção de deixar o emprego e a profissão^(17,18)^.

Estudo realizado no sul do Brasil avaliou a intenção de deixar a enfermagem durante a pandemia de COVID-19 em diferentes cenários e níveis assistenciais, com prevalência de 24,6% entre os participantes^(17)^. Na perspectiva internacional, estudo suíço encontrou uma prevalência de 55% de profissionais de enfermagem que pensaram em abandonar suas carreiras durante o período da pandemia de COVID-19^(18)^. Estudo de revisão sistemática e metanálise identificou que aproximadamente um terço dos profissionais de enfermagem que atuaram durante a pandemia de COVID-19 apontou intenção de deixar o emprego^(19)^. Fora do contexto da pandemia, no período pré-pandêmico, estudo conduzido em 40 hospitais na Itália encontrou que 35,5% dos enfermeiros tinham o desejo de deixar a profissão devido a fatores como exaustão emocional, elevada relação paciente/enfermeiro e falta de realização pessoal^(20)^.

A prevalência mais baixa de intenção de deixar o emprego neste estudo pode ser explicada pelo tipo de vínculo laboral do tipo concurso/seleção pública nas instituições participantes, o qual confere estabilidade empregatícia aos concursados e compõe uma equipe qualificada para o exercício das funções, possivelmente contribuindo para sentimento de realização profissional. Além disso, a remuneração praticada nessas instituições é superior aos salários verificados no mercado nacional^(15)^, acrescida ao prestígio e oportunidades de desenvolvimento profissional relacionados ao vínculo em hospital federal de ensino, fatos que podem justificar a baixa intenção de deixar o emprego nos cenários da pesquisa.

Nesta investigação, a intenção de deixar o emprego foi associada à idade, sendo mais prevalente entre os trabalhadores mais jovens (*p*=0,014) e com menor tempo de experiência no cargo (*p*=0,046). Estudos de revisão sistemática apontaram a idade como um dos fatores associados à intenção de deixar o emprego, sendo que os trabalhadores mais jovens têm maior intenção de deixar o emprego^(9,21)^. Estudo desenvolvido nos Estados Unidos com 207.636 enfermeiros identificou uma prevalência de 21% de intenção de deixar o emprego, sendo que a chance de referir tal intenção reduziu 1,5% a cada ano de experiência no emprego. Neste mesmo estudo, os profissionais com idade entre 22 e 37 anos referiram maior intenção de deixar o emprego, quando comparados aos demais, com exceção daqueles entre 73 e 90 anos, devido à intenção de aposentadoria^(22)^.

Neste estudo, os profissionais de enfermagem referiram sentir que a equipe está quantitativamente inadequada em mais da metade do tempo de trabalho e teve maior intenção de deixar o emprego (*p*=0,026). A sobrecarga de trabalho resultante da insuficiência de pessoal, recursos materiais, alta demanda de pacientes, gestão e organização da unidade pode afetar mentalmente e fisicamente a equipe de enfermagem, impactando diretamente a segurança do paciente^(23)^ e a intenção de deixar o emprego e a profissão^(1,21)^. O enfermeiro, como líder da equipe de enfermagem, deverá articular, junto aos gestores, a melhor organização quanti-qualitativa da equipe de enfermagem, considerando a complexidade e multidimensionalidade das atividades desenvolvidas no contexto assistencial e gerencial^(24,25)^, com foco na qualidade assistencial e segurança do paciente^(26)^.

A escassez de profissionais de enfermagem é um problema crescente no cenário global e que pode se agravar nos próximos anos devido à elevada intenção de deixar a profissão. Uma preocupação adicional em países em desenvolvimento se refere à tendência de movimentos migratórios de enfermeiros para países com melhores condições econômicas, comprometendo a quantidade de profissionais disponíveis em áreas mais vulneráveis^(27)^. Além disso, a perda de talentos, ou *brain drain,* representa um movimento crescente na enfermagem com possíveis consequências relacionadas à perda de profissionais qualificados e aumento da carga de trabalho dos profissionais remanescentes, enquanto os migrantes podem experienciar dificuldades relacionadas à adaptação ao país de destino e vivenciar xenofobia^(28)^.

Neste estudo, predominou o relato de satisfação com o cargo atual, conforme foi observado em outra investigação^(29)^. Esse resultado é diferente do observado em outros estudos, nos quais a satisfação foi menos prevalente entre os profissionais de enfermagem. Estudos realizados durante a pandemia de COVID-19 identificaram alta satisfação no trabalho em 11,6% dos enfermeiros no Irã^(30)^ e em 25,2% dos enfermeiros no Egito^(31)^. Estudo brasileiro identificou satisfação entre 26,9% dos participantes com prevalência de sentimentos de ambivalência no que se refere à satisfação (72,1%)^(32)^. O medo da doença e as altas cargas de trabalho influenciaram a satisfação com o cargo dos profissionais de enfermagem na linha de frente da COVID-19 nas Filipinas, aumentando a intenção de deixar o emprego^(33)^. Cabe mencionar que, na concepção de satisfação, critérios distintos são percebidos por cada trabalhador.

Apesar disso, a baixa satisfação laboral foi associada à intenção de deixar o emprego nesta investigação (*p*<0,001). A intenção de deixar o emprego foi três vezes maior entre os profissionais que referiram insatisfação com o cargo atual (OR=3,519; IC=1,594-7,766; *p*=0,002). A insatisfação no trabalho foi apontada como um dos fatores relacionados à intenção de deixar o emprego e a profissão em diferentes estudos^(1,21,34)^.

A baixa disponibilidade de profissionais de enfermagem representa um sério risco ao cumprimento dos ODS^(27,35)^, não somente na meta três, explicitamente relacionada à saúde, mas em todos os demais, visto que a atuação da enfermagem tem interface ampla com os ODS^(4)^. Estratégias capazes de melhorar a retenção dos profissionais de enfermagem incluem reduzir a carga de trabalho, melhorar condições de trabalho, investir em planos de carreira e melhor remuneração, melhorar o dimensionamento de pessoal, promover a liderança da enfermagem, garantir suporte social, e favorecer o equilíbrio entre vida pessoal e trabalho^(34)^, promovendo, dessa maneira, maior satisfação no trabalho.

### Limitações do estudo

Esta pesquisa apresenta limitações, uma vez que está inserida no contexto hospitalar universitário federal, no qual a estabilidade e a remuneração são fatores que podem impactar a intenção de deixar o emprego. Somam-se a isso as limitações do desenho de pesquisa transversal que apresenta apenas um recorte temporal do fenômeno estudado. Dessa forma, recomenda-se a replicação deste estudo em outros contextos e momentos, como também em diferentes níveis de assistência.

Além disso, a pandemia de COVID-19 foi um marco que expôs as fragilidades dos sistemas de saúde em todo mundo, refletindo na importância da organização do trabalho em saúde. Foi um momento de cuidados exaustivos que impactaram diretamente a satisfação e intenção em deixar a profissão, determinando a necessidade de novos estudos sobre a temática e o desenvolvimento de políticas públicas de incentivo e valorização dos profissionais de enfermagem.

### Contribuições para as áreas da enfermagem, saúde ou políticas públicas

O presente estudo apresenta dados relevantes, e traz à luz um tema emergente para a enfermagem brasileira, que poderá em poucos anos vivenciar a escassez de profissionais. Nesse sentido, identificar enfermeiros e técnicos de enfermagem que pretendem deixar seus empregos e a profissão sinaliza para a liderança de enfermagem e para os gestores de serviços de saúde a necessidade de (re)examinar os potenciais fatores associados a esse problema.

Vale destacar que a investigação realizada em três hospitais brasileiros de diferentes regiões do país aponta a variáveis determinantes em comum, indicando a necessidade premente de se debater sobre políticas públicas de pessoal de enfermagem, como o investimento em melhores condições de trabalho, valorização e reconhecimento da profissão, formação continuada, entre outras. Acredita-se que, nessa direção, será possível promover a retenção da força de trabalho de enfermagem, considerada um dos pilares fundamentais para garantia de saúde à população com qualidade e segurança.

## CONCLUSÕES

Esta investigação apontou que a insatisfação no trabalho pode aumentar em até três vezes a chance de intenção em deixar o emprego. Entre os participantes do estudo, a intenção de deixar o emprego foi associada aos profissionais mais jovens, com menor tempo de experiência no cargo, formação em nível superior ou pós-graduação, insatisfeitos no trabalho, avaliando como inadequado o número de profissionais de enfermagem na maior parte do tempo trabalhado. Ainda que o percentual de intenção em deixar o emprego neste estudo esteja abaixo daqueles identificados em estudos internacionais, destaca-se a crescente preocupação com os possíveis déficits de profissionais de enfermagem no Brasil e com a migração de talentos da enfermagem brasileira para o exterior. Destaca-se a necessidade de ampliação dos estudos nacionais sobre a temática, a fim de identificar no cenário nacional a intenção de deixar a enfermagem, bem como a importância de investimento para a retenção desses profissionais no país.

## Data Availability

Os dados de pesquisa só estão disponíveis mediante solicitação.
